# Community nurses’ perspectives on a novel blended training approach: a qualitative study

**DOI:** 10.1186/s12912-022-00893-3

**Published:** 2022-05-12

**Authors:** Chou Chuen Yu, Khanh M. Le, James A. Low

**Affiliations:** 1 Geriatric Education and Research Institute Ltd, 2 Yishun Central 2, 768024 Singapore, Singapore; 2 Department of Geriatric Medicine, Khoo Teck Puat Hospital, 90 Yishun Central, 768828 Singapore, Singapore

**Keywords:** Community nursing, Nursing training, Training evaluation

## Abstract

**Background:**

Transiting into the community setting often presents novel difficulties for nurses because the role demands skills that might not have been obtained through usual clinical experience or training. The Ageing-in-Place Community Care Team (AIP-CCT) Community Nurse Basic Training programme was developed to address this learning gap. This training programme prepares nurses to lead in a multi-disciplinary team in delivering patient-centred care to patients with progressive or life-limiting conditions in the community setting. This study evaluated the inaugural training programme provided to a group of nurses from an acute hospital in Singapore.

**Methods:**

Qualitative in-depth interviews were carried out with 13 participants from the training programme three-months after completion of the AIP-CCT Community Nurse Basic Training programme provided by an acute hospital to understand the programme’s impact on their knowledge, skills and clinical practice, as well as barriers and facilitators to learning.

**Results:**

Overall, perception towards the training course was mixed. Course content was found to be relevant, and participants reported that training led to improvement in their practice. However, experienced nurses felt that the content of some modules were lacking in depth. This could have explained why only junior nurses tended to hold favourable attitudes and felt that the training led to increase in their confidence level. Although medical content was assessed favourably, the course was not able to address some of the constraints faced by community nurses such as managing expectations and handling difficult patients in the home care setting. For some modules, face-to-face training was preferred and e-learning components can be improved to increase communication and interaction.

**Conclusion:**

This study provided insights into how a community nurse training programme could be developed to meet the needs of community nurses. The training was able to reinforce skills and knowledge, address knowledge gaps and provide new clinical care approaches and communication strategies. These incremental effects on experienced community nurses could be extrapolated to have greater benefits for inexperienced community nurses. Based on findings of the study, potential changes to the training programme were discussed to improve training outcomes.

## Background

### The need for community nursing programmes

High readmission rates, often seen among the elderly, can lead to serious shortage of acute hospital beds in a rapidly ageing population [1]. This phenomenon can exert significant strain on limited healthcare resources and negatively impact quality of care. Factors contributing to high hospital readmission rates, apart from medical needs, include social, behavioural and environmental factors that may not have been adequately managed in the acute hospital setting [2, 3]. Various measures have been introduced to address these problems, including home visits, structured discharge planning, personalised health care programmes, enabling self-management, multidisciplinary care approaches, integration of case management, primary and social care, and telemedicine [4–9]. A local study by Toh et al. [10] also suggested that by alleviating caregiver stress, older patients’ period of hospitalisation could be shortened.

The Ageing In Place Programme (AIP) was initiated in 2011 in Singapore to tackle patient and caregivers’ social and nursing needs, which was able to successfully alleviate caregiver stressors [11]. It later merged with the transitional care service to form the AIP-Community Care Team (AIP-CCT) programme to reduce hospital readmission rates by addressing modifiable contributing factors, providing home visits and formulating individualised hospital care plans. The new nurse-led programme, supported by specialists and community partners, provided a location-based and multi-disciplinary team service that delivered patient-centred care to complex patients with progressive or life-limiting conditions. AIP-CCT community nurses visited newly discharged patients at their home for a period of between 3–6 months post-discharge. Care is guided by a comprehensive needs assessment, taking into account the medical, functional, nursing and psycho-social profiles of the patient.

### Challenges faced by community nurses

Working in the community setting, community nurses’ roles are drastically different from the clinical settings they have trained and worked in [12]. The challenges include working independently and taking leadership roles in the management of cases; being resourceful; having broad-based and specialised knowledge to make accurate comprehensive assessments; and having excellent communication skills to convey information and coordinate community assistance services for patients. Additionally, their job also requires performing healthcare education, case management, and end-of-life care. As cases are both medically and socially complex, community nurses are required to adapt approaches and procedures on the spot, respond to environmental, physical and social hazards in patients’ homes, and assess caregiver burden to provide appropriate care advice [13, 14]. Beyond the clinical challenges, they also often face communication barriers and complexities in nurse-patient relationships [13–15].

Community nursing often demands cognitive skills, including assertiveness, counselling, critical thinking and evidence-based practice [16, 17], which may not have been obtained through clinical experience or training [16]. Many community nurses reported feeling ill-equipped for practice even with specialised training [17–19], and prior studies suggests that it takes between 6 to 9 months for newly qualified nurses to feel comfortable working in the community [19, 20].

## Study settings and aims

To address challenges faced in transitioning to the community setting, the AIP-CCT Basic Community Nurse Training Programme was developed and tailored at a regional acute hospital in Singapore, to equip nurses across all staff grades and background with competencies necessary to provide care for homebound patients. This study aims to understand the learning experience of community nurses attending the training. In addition, it explores barriers and facilitators to learning and translation of knowledge into practice within a community care setting, based on findings from the first training of AIP-CCT Basic Community Nurse Training programme. By understanding the needs of community nurses, future training curriculum and delivery modality could be improved.

## Method

### Study design and participants

This study employed an evaluation research design using a phenomenological method to qualitatively describe and understand the experiences of the programme from the perspective of CCT nurses who have gone through it both in terms of *what* was experienced and *how* it was experienced [21]. Data was collected through in-depth interviews and a semi-structured interview guide was used to facilitate participants’ sharing based on Kirkpatrick’s Model [22]. Questions are categorised into four areas: *Reaction* (e.g. “How do you feel about the course in general?”), *Learning* (e.g. “How has the course changed your understanding or enhanced your skills?”, “How has the course changed the way you feel about your work?”), *Behaviour* (e.g. “How have you applied what you learnt at work?”) and *Result* (e.g. “How has the course helped you to addressed the challenges you face at work?”). In addition, the interview asked about participants’ work experience as community nurses (“What do you find challenging about your work?”), facilitators and barriers to learning (“How did the course format, facilitators, materials, etc. support or challenge your learning?”), and possible improvements (“How can the course be improved to support your learning?”).

Purposive sampling method was used where all of the community nurses attending the first AIP-CCT Basic Community Nurse Training programme were invited to participate in the study. It is worth noting that the first training was carried out to assess its effectiveness as well as to fill in knowledge and skill gaps of current community nurses; therefore, the training programme was conducted to all community nurses in the organisation at that point in time, regardless of their level of experience. In total, thirteen participants agreed to be interviewed. On average, participants had many years of working experience as senior staff nurses and have had experience working as community nurses even prior to attending this formal training programme. The demographic profile of the participants is shown in Table 1. Informed consent was taken from all participants.

**Table 1:** Participants demographic information

Characteristic	*N* = 13 (100%)
Age	*M* = 43.1; *SD* = 11.21
Sex	
Female	13 (100%)
Race	
Chinese	8 (61.5%)
Malay	2 (15.38%)
Indian	3 (23.08%)
Seniority level	
Staff Nurse	1 (7.69%)
Senior Staff Nurse	11 (84.62%)
Others	1 (7.69%)
Number of years of nursing experience	*M* = 14.54; *SD* = 13.19
Number of years of community nursing experience	*M* = 2.85; *SD* = 2.16

The study received ethics approval from the local healthcare group’s Institutional Review Board, i.e. The National Healthcare Group Domain Specific Review Board (Reference number 2010/00020).

### The AIP-CCT basic community nurse training programme

The training programme was conducted between 2017 and 2018. The training curriculum included eight modules that covered the prescribed standards and competencies (Table 2). The course employed a blended learning approach combining 15 h of online lectures and 27 in-person teaching hours that spanned 14 weeks. The online component included interactive video lectures, demonstrations, and assessments. During in-person training, participants attended faculty-facilitated, learner-centred workshops that consisted of demonstrations, hands-on practice, case discussions, simulations, quizzes, and on-the-job training (e.g. home visits, multidisciplinary meeting, clinical attachment, etc.). These multiple learning strategies were utilised to allow for a targeted, individualised, and experiential learning experience while being flexible and suitable for working professionals. Healthcare professionals from various disciplines were invited to facilitate training and discussions.

**Table 2:** Training curriculum

Module 1: Community Care Team Overview	1.1 Introduction to Community Care Team (CCT)
	1.2 Basic Elements of AIP-CCT
	1.3 Community Care Team Process Briefing
Module 2: Assessment, Care Planning and Coordination	2.1 Needs assessment
	2.2 Home visit assessment
	2.3 Physical examination workshop
	2.4 Care planning and coordination
	2.5 Home visit with supervision
	2.6 Care planning and MDM
Module 3: Interprofessional CCT Management	3.1 Overview of regional health system
	3.2 Community services
	3.3 Financing schemes for patients
	3.4 Psychosocial support for CCT patient case discussion
	3.5 Functional and home assessment
	3.6 Mobility and exercise
	3.7 Occupational therapy workshop
	3.8 Physiotherapy workshop
	3.9 Dental hygiene
	3.10 Dysphagia
	3.11 Nutrition
	3.12 Speech therapy workshop
	3.13 Dietetics case discussion
	3.14 Medications and polypharmacy
	3.15 Wound care
	3.16 Medication reconciliation quiz
	3.17 Wound care quiz
Module 4: Chronic Disease Management	4.1 Diabetes mellitus
	4.2 Heart Failure
	4.3 Diabetes Mellitus workshop
	4.4 Heart failure workshop
	4.5 COPD
	4.6 Stroke
	4.7 COPD workshop
	4.8 Case discussion: Going home after stroke
Module 5: Geriatric Care	5.1 Overview of care of geriatric patients
	5.2 Dementia
	5.3 Dementia and BPSD management
	5.4 Dementia case discussion
	5.5 Continence care
	5.6 Continence workshop
	5.7 Falls
	5.8 Recurrent fall case discussion
Module 6: Palliative Care	6.1 Basics of palliative care
	6.2 End-of-life care at home
	6.3 Case discussion
	6.4 Advanced care planning (ACP) clinic attachment (Certified ACP facilitators only)
Module 7: Nurse-Client Relationship	7.1 Enabling self-management
	7.2 Handling self
	7.3 Motivational Interview
	7.4 Case discussion: Tackling tough love
Module 8: Health Informatics and Technology	8.1 Health informatics
	8.2 Telehealth
	8.3 Telehealth communications

### Procedures

Trainees were informed of the study aims and procedures on the first day of the training and were invited to participate in the study. Three months after completion of training, trainees who accepted the invitation participated in an in-depth face-to-face interview, with sessions lasting approximately 60 min and conducted at the researchers’ institution at a time chosen by the participant.

The interviews were conducted by the first two authors, a female full-time researcher with a bachelor’s degree in psychology and a male full-time researcher with a doctorate degree in psychology. Both researchers had experience conducting research on education needs of healthcare professionals working with older population and had no prior relationship with the participants. Participants also did not contact the research team prior to study commencement. With regard to probing techniques, MK and CC approached the interviews from an outsider “naïve” position, thereby reducing the possibility of biasing the responses.

### Data analysis

Verbatim transcripts of interviews were first cross-checked against the recording to ensure accuracy. Directed qualitative content analysis was used to analyse the data. The authors first read through transcripts multiple times to stay immersed in the data. Transcripts were then coded manually and thematically analysed by the first author. To augment the analysis of the transcripts, field notes containing summaries of participants’ responses to the interview questions were taken during the interviews. The interviews were audio-recorded and transcribed verbatim by professional transcribers. These procedures in place adhered to common best practices on academic rigour to ensure trustworthiness in qualitative research.

Three participants refused to be audio recorded, therefore, field notes of these interviews were coded instead of verbatim transcripts. The codes were categorised deductively based on the areas explored by the interview guide (i.e. reaction, learning, behaviour, result, learning experience) and a preliminary list of themes was developed. This accounted for the “directed” part of the analysis based on the a priori questions formulated using the Kirkpatrick’s Model. Additionally, discussions and views shared outside of the topic guide were generated using inductive coding to enhance the understanding of the experiences of participants going through the training curriculum in addition to work experience as community nurses. The codes and preliminary themes generated were reviewed by the second author. The final themes were discussed and agreed upon by both authors. Following Francis et al. [23], data is considered to reach saturation as no new theme was generated in the last three interviews.

## Results

### Course content relevant to community nursing

In-depth interviews with participants suggested that the course covered a sufficient number of topics which were on the whole relevant to community nursing (see Table 2 for the list of topics). However, given that majority of participants were experienced community nurses, they were familiar with most of the topics covered. Hence, the training was often considered as a refresher for previously acquired knowledge. Notwithstanding this perspective, many of the participants recognised the relevance of the topic for new community nurses:

“For people without community experiences, I think that is actually quite a good depth of knowledge for them to start.” – P05

Moreover, the training was reported to provide a structured care framework and approach to community nursing and address pre-existing knowledge gaps:

“Most of the things that we learn in the community is on the job training. Because you are alone so you decide there and then what you want to do. Then after that whatever decision or intervention that you decide, just call the doctor for consultation. But this module, the one that we went through, is really structured, structured then (so) , of course, (it) equip(ed) the nurses (with) knowledge.” – P01

It should however be pointed out that the view on the relevance of the topics was not homogenous and some participants shared that the course was actually too content-heavy and may not be necessary especially for new community nurses who may only come to understand or appreciate them at a later stage as they acquire more practice experience. Others, although agreeing the topics were relevant, felt that better calibration was needed as some of the content they had to go through was “boring” or unnecessarily detailed.

For many, better calibration of topics included having more rather than less content. This led to the perception by some that the emphasis of the training was on breadth rather than depth and perhaps a longer duration for the course was needed so that the issue of depth in training can be addressed. Experienced community nurses expressed that important topics were either missing or should have been more in-depth. These included topics such as symptoms of issues commonly seen in the community, palliative care, dementia, etc. A list of topics that have been suggested to be included or covered in greater depth is shown in Table 3. These topics were suggested as issues encountered in the community were often complex and entailed high level of skills:

“[…] what the course taught us is pretty basic. But what we are expected to assess outside is even more” – P03

**Table 3:** Topics suggested by participants to be included in the course

Necessary topics to be included:	Participants’ topics of interest:
• Managing/communicating with patients and family members • Referral points • Mouth exercises • Symptoms of issues commonly seen in the community • Palliative care • Chronic illnesses • Medication • Behavioral issues • Blood taking • Social issues • Motivation interview • Psychosocial issues • Dementia & BPSD	• Rehabilitation • Palliative care • Physical examination • Chronic illnesses • OSCE • Pathophysiology • Continence • Swallowing assessment • Motivation interview • Wound care • Medication • Diabetes • Speech therapy • Asthma • Heart failure • COPD

### Training led to improvement in practice

Although participants were experienced nurses, they reported that the training improved their practice as they got to be exposed to new areas of care and interacted with specialists to clarify questions that they had (e.g., P04 and P05 sessions with dietician and specialist nurse on dementia). Perhaps more importantly, the exchanges that participants had with other nurses with different practice experiences facilitated learning. As participant P06 mentioned, this interaction with other course mates led to an exchange of questions and thoughts related to practice. Another participant (P11) reported that such interactions facilitated by instructors served to increase the motivation to learn beyond helping to clarify on doubts. Experienced nurses also benefited from the tools and frameworks equipped to them by the course. For instance, one participant (P11) reported that prior to the course, her approach might have been more intuitive whereas after the training she was better equipped to address issues in a more systematic manner.

The course was not without its flaws. One of the biggest obstacles faced by this group of community nurses seemed to be non-clinical in nature and the course did not serve to adequately address some of these challenges in content or solution. Among them, language posed the biggest challenge as they encountered patients or caregivers who spoke different languages. The use of interpreters could make it harder to connect and build trust when communicating. Other common challenges included managing expectations of patients and family members, handling difficult and uncooperative patients and family members, and dealing with behavioural, emotional, and social issues of patients. These challenges faced in the community home care setting were not adequately addressed by the course.

“Challenges in CCT actually is mainly, I think, is the social component for the patient because if the patient’s social support is not there, so even (if) we are also supporting the patients, the condition we know becomes more complex because there’s really, social issues. But if social support is very good then even (if) medical conditions is very bad, they also can handle very well. So, the social component is quite (a) challenge.” – P10

Notwithstanding this inadequacy, participants were mindful that these challenges faced were complex in nature and could not be addressed by this or any courses for that matter. Often, they learn to handle such challenges through experiences on the job. Participants acknowledged, however, that their practice did improve due to what they have learnt about better understanding of patients’ behaviours and personalities and communication strategies with them:

“[…] then maybe (I) have, like better understanding in terms of the patient, the so called the process, maybe how come they would not want to change who they are, the management of their condition and those things. Because sometimes it’s due to the personality of the patient and those things.” – P07

Other communication strategies learnt pertain to gathering and presenting information more effectively and efficiently to other team members:

“I find that, with this SBAR thing especially, we are more focussed. I know what I want to know from the patient. More direct. Even when I want to pass the message or discuss the case with the doctors, by using this, (it) is more focussed so the doctor will know…..what actually happened or what the situation at that point (is), what is the background of the patient and (what has) actually happened to the patient.” – P02

### Positive attitudes and confidence pre-and-post training

Participants in general believed that the job was meaningful and were confident about performing community nursing even before the course. This was the case as most of the participants were senior nurses with experience in the community and therefore felt that the course did not significantly alter their existing knowledge, skills, or abilities. However, some participants reported feeling more confident working in the community following the training as it addressed knowledge gaps and helped revise their clinical skills and knowledge. For instance, participants mentioned that their communications skills have improved, and one participant illustrated about how she was able to apply the knowledge gained from the course and present a case to a doctor with confidence (P08). One participant (P11) remarked that although the course did not change her confidence, she felt this would have been so for new community nurses without the experience. Another participant (P02) mentioned that the training would be beneficial for new community nurses since they were less likely to need to rely on prior encounters and experiences. The latter refers to the concept of practical wisdom where one learns through personal experience and encounters. In other words, one does not have to learn through one’s own experience but through the experiences of others (the trainers).

### Preference for in-person training

Participants felt that there was a good mix of e-learning and in-person training, which increased engagement from learners:

“I think they are good, because I think it caters to different learners’ need. Some people are good in role play, so let them act out.” – P05

E-learning allowed for the flexibility crucial to working professionals. Participants could study at their convenience, with the possibility of reviewing lessons if needed. However, a few participants shared that e-learning sessions were content-heavy, and questions could not be clarified immediately, which impeded knowledge absorption:

“If you’ve got, I mean the interest and the energy, then you can listen very carefully. But if let’s say in the late afternoon maybe some listen to the same audio for 10 minutes, we’re going to sleep already, like children they go like that. It’s not interactive and then, it’s really like, the information go in or not, I’m not sure.” – P10

Heavy workload was cited by most participants as a barrier to learning. Despite having protected time during work hours for e-learning, some participants had to forgo self-study time or tried to complete learning materials in haste given their pressing work demands. This issue was further compounded by the e-learning deadlines which, if exceeded, would deny participants continued access to the training material:

“[…] because we did it during our working hours right, so our main focus when we’re doing the e-learning is more of finishing the module instead of keeping it in my memory or recall it.” – P04

Participants showed a strong preference for face-to-face or in-person training for some of the modules. Participants felt that topics were better explained and easier to understand while questions could be clarified immediately. Moreover, a fixed timing and study space avoided interruptions and allowed more focus on the lesson. In addition, participants could interact with each other and through case discussions in class, nurses, regardless of their seniority, could learn from others as they listened to different approaches to care:

“Because there’s interactive (interaction), and then there is…because when you share your case, it’s not only your own experience, it’s actually other groups, other people, what they, if let’s say they encounter this, what is their option of care? What is the opportunities? So you can take in and then you can give your own and from there, you know, discuss. And then you know something new, that’s one thing.” – P07

## Discussion

In general, participants felt positive about the work and were confident performing the required tasks. The training was able to reinforce previously learnt skills and knowledge, fill knowledge gaps, and provide new clinical care approaches and communication strategies. These positive outcomes on already experienced community nurses demonstrated the training’s potential to enhance the competencies of inexperienced community nurses even more. An important contribution of this study was the identification of the barriers and enablers to learning for community nurse training. By collating feedback from the trainees, this study provided valuable insights into how a community nurse training programme could be developed to meet the needs of the modern-day community nurse who has to deal with the complex nursing and medical problems of patients, especially in an ageing society.

### Training content adequate for new community nurses

The areas covered in the training were adequate for new community nurses, providing them with an overview and summary of important topics crucial for the role of community nurses. However, different concerns were raised about the depth of training. As mentioned in the findings, experienced community nurses in our sample felt that the training did not alter their existing attitude or boost their confidence given that the training felt more akin to a refresher course. Beliefs and attitudes of participants in this sample toward community nursing would already have been formed and fixed prior to attending the course and therefore it is somewhat expected that the course would not be able to change attitudes in a big way for this group. Taking into account the aim of the training, which is for the on-boarding and setting of service standards of community nurses, the broad range of topics considered important for community nurses, and the time constraints of learners and their employers, it might be necessary to develop and incorporate a separate advanced training programme for these community nurses with some years of work experience. Despite this limitation, it was also apparent that having community nurses of different practice experience coming together for the course is important for informal learning. Tacit learning can occur in such instances [24–26]. As shared by the participants, interaction amongst nurses of different background allowed informal learning to take place that supported the intended curriculum whereby learners were able to reflect critically on the activities, norms and shared understanding of community nursing work. Regarding the adequacy of the existing programme, it would not be feasible to address all non-medical issues community nurses encounter at work due to their complexity. Hence, the training programme could include components directing new nurses to the appropriate and related training resources.

In addition, the study pointed out that the biggest challenges facing community nurses were mostly non-clinical in nature, which was in line with findings from a previous study [13]. The AIP-CCT training programme focused heavily on the clinical aspects of care and, hence, was not able to fully address the difficulties related to psycho-social issues. To train community nurses in communication and inter-professional skills, curriculum developers should appreciate and establish clear roles for community nurses vis-a-vis other members of the multidisciplinary team, setting a common goal for the team and building conflict resolution skills for community nurses [27].

### Benefits of blended learning approach

It is believed that blended learning could bring greater benefits to the learner compared to traditional or non-blended pedagogies [28]. The combination of online and face-to-face training was generally well received by our participants but many felt that having more face-to-face sessions would be beneficial. While current literature suggests that there is little difference between the impact of e-learning and traditional teaching methods such as didactic lectures [29], participants in this sample preferred face-to-face training and various reasons were given by participants to explain the preference. The flexibility provided by e-learning at one’s own time and discretion was on the contrary not beneficial as community nurses are faced with heavy workload and this meant there was no protected time given for learning. This finding was in line with previous studies which indicated that work commitment and heavy workloads are barriers to nurses’ participation in education programmes [30, 31]. Adjusting the mix and proportions of e-learning and face-to-face training might increase knowledge dissemination and absorption. As suggested by the participants, e-learning could be used for imparting theoretical knowledge while practical skills could be reserved for in-person face-to-face training. Alternatively, online modules can be redesigned to permit active real-time communication and interaction, not only amongst students but also between students and instructors. Online communication and interaction are valuable as it would permit students to encourage and motivate each other, as they would in-person, and allow questions to be addressed by instructors in real-time. This has been demonstrated in research that has shown that being able to interact with peers or medical educators plays an important part in the success of online learning [32, 33]. Whilst replacing e-learning component of the course with face-to-face training would possibly address some of the motivational concerns faced by some participants, this is not practical as it would be resource intensive and further extend the duration of the course. The current long-drawn COVID-19 pandemic has also shown that remote training is critical for healthcare professionals given the various safe distancing restrictions imposed in hospitals [34]. With a redesign of the online modules to allow greater interaction and collaboration, the e-learning component could be highly relevant and important for training of new initiates and the continual education of existing community nurses.

### Limitations

The small sample size in the quantitative arm of the study limits the inferences that can be made to the wider nursing population involved in community nursing. Participants seemed reticent at the beginning of the interviews, possibly due to the small size of the working team which led to concerns over anonymity. Their concerns were assuaged after being assured of confidentiality and that the study team was independent of the training team. Nonetheless, considering that the majority (72.22%) of our community nurses were interviewed in-depth, data saturation was achieved and could be presumed to reflect the views of the population of community nurses in the hospital. As the course was concluded in 2018 and data collection and analysis occurred thereafter for over a period of two years, one other limitation pertains to the temporal relevance of the findings. However, as the practice of community care and training of community care nurses tend not to change drastically, findings on the training content continue to be applicable and relevant, granted training content will need to be updated in view of shifts in needs of the community. Moreover, the importance of community care is even greater in the pandemic situation, where patients are kept at home and away from hospitals, day care centres and other care facilities. The aging population and shortage of acute hospital beds also mean more people will require community services in the upcoming years, not only in Singapore, but also in other countries still in the nascent stage of developing their community services and the need to train their healthcare staff to run these services remains pressing. Notwithstanding the temporal limitations, findings will benefit other countries or healthcare systems planning to design a course relating to community services.

## Conclusion

This study showed the potential of a customised blended training program in assisting community nurses with their duties. Encouraging results from experienced community nurses indicates that the course could have a greater impact on new community nurses. Data collected also sheds light on important issues such as work requirements and the level of demand for knowledge required in community nursing. Modifications based on the findings of this study, namely on the choice of training topics, delivery modality, and training logistics will help to improve the training’s effectiveness in the future.

### Future directions

This study presented experienced community nurses’ perspectives on a basic training programme for new nurses. While proposing possible improvements to the course, this article suggests that the programme has positive influence on community nurses’ practices. Future studies evaluating new community nurses experience toward the training and their experiences in the community will be beneficial. In addition, future studies can look into the economic benefits of this training programme on the overall healthcare system. With the population ageing rapidly and the bulk of healthcare budgets being spent on the older population, bringing healthcare from hospitals to the community would relieve pressure on the system, cut down on medical expenses, and improve quality of care.

## Data Availability

The datasets used and/or analysed during the current study are available from the corresponding author on reasonable request.
